# Gap in measles vaccination coverage among children aged 9 months to 10 years in Ho Chi Minh City, Viet Nam, 2014

**DOI:** 10.5365/wpsar.2017.8.2.001

**Published:** 2019-12-28

**Authors:** Hoang Quoc Cuong, Ho Xuan Nguyen, Pham Van Hau, Nguyen Le Khanh Ha, Phan Trong Lan, Anthony Mounts, Tran Minh Nhu Nguyen

**Affiliations:** a Pasteur Institute, Ho Chi Minh City, Viet Nam.; b Field Epidemiology Training Program, Ho Chi Minh City, Viet Nam.; c World Health Organization, Viet Nam.; d Centers for Disease Control and Prevention, Viet Nam.

## Abstract

**Introduction:**

When Viet Nam launched the Expanded Programme on Immunization in 1981, it covered six vaccines, including measles. Subsequently, Viet Nam experienced a marked reduction in measles infections. A nationwide measles epidemic occurred in April 2014 and an investigation found that 86% of affected children aged 9 months to 10 years were not fully vaccinated; therefore, understanding the reasons for not vaccinating could improve vaccination coverage.

**Method:**

We performed a cross-sectional study to determine vaccination coverage and reasons for non-vaccination among children aged 9 months to 10 years in six districts in Ho Chi Minh City with the highest number of measles cases in 2014. Measles vaccination status of the youngest child in each household was determined and reasons for non-vaccination were investigated. A χ^2^ test and multiple logistic regression were used to identify independent predictors of full vaccination.

**Results:**

In total, 207 children were enrolled during the study period in 2014. Full measles vaccination coverage was 55% in these households, and 73% of parents were aware of the importance of measles vaccination to protect their children. We found that the father’s education level (under high school versus high school and above) and the site where the survey was conducted were significantly associated with vaccination status.

**Conclusion:**

The vaccination coverage was lower than the coverage reported by district preventive medicine centres of the seven study wards. Lack of the second vaccination was a key obstacle to eliminating the vaccination gap. A catch-up mass vaccination campaign or health promotion of measles vaccination directed towards parents should be considered to improve vaccination coverage.

The World Health Organization (WHO) has developed plans to eliminate measles in the Western Pacific Region, which includes Viet Nam. (*1*) However, recent measles outbreaks throughout the world, including in the United States of America, the Netherlands, Australia, China, the Philippines, Indonesia and Viet Nam have highlighted the challenges in achieving this goal. (*2*-*5*) In May 2014, more than 3900 confirmed measles cases and 133 deaths were reported in Viet Nam, a large increase in cases compared to 2012 (637 cases) and 2013 (1233 cases). (*6*-*8*) The Ministry of Health in Viet Nam introduced the Expanded Programme on Immunization (EPI) in 1981 with the support of WHO and the United Nations Children’s Fund (UNICEF). EPI provides immunization services through community health centres (CHCs) that dedicate one or two days per month to this service. In 2009, measles vaccination was administered at the ages of 9 months and 6 years; in 2011, the second dose administration was brought forward to 18 months of age. If children miss any of the measles vaccine doses, immunization services are tasked to administer the missed dose. Viet Nam has conducted periodic measles vaccine campaigns targeting children aged 9 months to 10 years at CHCs to address gaps in coverage among young children; however, according to the 2014 outbreak report, 82% of measles cases occurred in children under the age of 10, and 86% of the infected children were not fully vaccinated or had unknown vaccination status. (*9*, *10*) The proportion of the measles cases occurred in persons known to have no previous measles vaccines was 3%. The proportion in those who had only one dose was 3%. The proportion in those with unknown vaccination status was 6%

Ho Chi Minh City (HCMC), the largest municipality in Viet Nam, is subdivided into 19 urban districts and five rural districts. Urban districts are further divided into wards and rural districts into towns and communes. HCMC covers an area of 2061.4 km^2^ with a population of about 8.6 million people. With a population density of 4.2 persons per km^2^, there is a high risk of infectious disease transmission. (*11*) In 2013–2014, an outbreak of measles occurred in southern Viet Nam; a total of 3486 cases were reported, including 1023 cases in HCMC. This outbreak started in HCMC and spread to neighbouring provinces. (*10*)

The aims of this study were to describe measles vaccination coverage among children aged from 9 months to 10 years living in HCMC and to identify individual factors associated with and reasons for non-vaccination.

## Methods

### Study design and sample size

In June 2014, we conducted a cross-sectional study in the seven wards of HCMC with the highest number of measles cases, which were located in six different districts of HCMC (**Table 1**).

**Table 1:** Weighted vaccination coverage among children aged 9 months to 10 years in seven wards of six districts in Ho Chi Minh City, Viet Nam, 2014 (*12*)

Community	Number of children	Weight	Surveyed immunization coverage weighted by population			Reported immunization coverage weighted by population	
			Full (%)	Not full (%)	Full (%)		Not full (%)
**Ward 8, District 8**	**281**	**4.1%**	**5 (20.0)**	**20 (80.0)**	**216 (76.9)**		**65 (23.1)**
**Phuoc Loc Ward, Nha Be District**	**294**	**4.2%**	**18 (64.3)**	**10 (35.7)**	**268 (91.2)**		**26 (8.8)**
**Ward 7, District 6**	**361**	**5.2%**	**15 (60.0)**	**10 (40.0)**	**352 (97.5)**		**9 (2.5)**
**Truong Thanh Ward, District 9**	**490**	**7.1%**	**18 (62.1)**	**11 (37.9)**	**344 (70.2)**		**146 (29.8)**
**Binh Hung Ward, Binh Chanh District**	**1266**	**18.2%**	**14 (51.9)**	**13 (48.2)**	**997 (78.8)**		**269 (21.2)**
**Ward 4, District 8**	**1767**	**25.4%**	**14 (50.0)**	**14 (50.0)**	**1247 (70.6)**		**520 (29.4)**
**Linh Xuan Ward, Thu Duc District**	**2487**	**35.8%**	**17 (60.7)**	**11 (39.3)**	**2296 (92.3)**		**191 (7.7)**
**Total**	**6946**	**100%**	**101 (54.9)**	**89 (45.1)**	**5720 (82.4)**		**1226 (17.7)**

The formula to calculate sample size was: *n* = 4 (r) (1-r) (f) (1.1)/(e (*2*)) (p) (*n*_h_). (*13*) We planned to recruit 210 children into the study based on the estimated vaccination coverage (r = 98%) reported by the EPI in Viet Nam, taking into account the design effect (f = 1.5f), the proportion of children under 10 years old (*P* = 7.5%), the average household size (*n*_h_ = 4) and given α = 0.05 and 95% confidence interval. (*14*)

### Sampling and data collection

We selected study households using a cluster sampling method described by the Johns Hopkins Bloomberg School of Public Health. (*15*) Out of 259 wards with an average population of 24 000 people each (range: 10 000–61 000), the seven wards with the highest number of cases during the 2014 measles epidemic were selected. Questionnaires were collected in a designated facility in each ward, followed by randomly selected door-to-door visits to 30 households in each ward. We interviewed the parents or principal caregivers of the youngest child face-to-face using a standard questionnaire describing the child’s vaccination history. Vaccination status was determined by reviewing the child’s vaccination cards and through parental recall. The study excluded children who were not permanent residents of the ward. We confirmed the child’s residence status using household registration books. Lastly, we retrieved the previous year’s (2013) population-level vaccination coverage in the seven study wards from the district preventive medicine centres (DPMC).

### Data analysis and management

The analysis took into account the cluster design of the study. Study factors included demographic information such as age, gender, parents’ education level and employment; the number of children in each house; and the distance from the household to the closest vaccination site. We examined the relationship between study factors and vaccination status weighted by the number of children in each age group residing in each ward. Vaccination coverage was categorized into three groups: no vaccination, one-dose vaccination and two-dose vaccination. We then created a variable that reflected whether a child was fully vaccinated and used it as the outcome with two levels: fully vaccinated and not fully vaccinated. Fully vaccinated was defined as a child who either (1) was aged 18 months or older and had received two doses of measles vaccine or (2) was aged 9–18 months and had received one dose of measles vaccine. Not fully vaccinated was defined as partial or non-vaccination, including children of any age who had either not received any measles vaccine or children aged ≥ 18 months old who had received only one dose of measles vaccine. The study population for the analysis also included the children ≥ 18 months of age who did not receive at least one dose of measles vaccine. A χ^2^ test, unadjusted and adjusted logistic regression models were computed by R statistical software (version 3.3.0, R Foundation for Statistical Computing, Vienna, Austria) to explore associated factors of vaccination. The R package BMA was used to conduct Bayesian model averaging approach, which not only accounts for the uncertainty in variable selection by averaging over the best models but also combines estimate and prediction. (*16*) For the multivariable analysis, variables were selected by univariate analysis of each variable using a p-value cut-off point of 0.25. Variables were also selected that were previously known to be important risk factors, such as parental awareness, parents’ level of education and fear of adverse reactions. Models with lower Bayesian information criterion and higher posterior probability are preferred. (*17*) Odds ratio (OR) and 95% confidence interval (95% CI) were used to identify the relationship between independent and dependent variables. Statistical significance was set at *P* = 0.05 to allow for the incorporation of model uncertainty into inference.

The survey was approved by the Pasteur Institutional Review Board (No: 272/PAS-QĐ on 20 June 2014).

## Results

Of the 210 children recruited for enrollment, 207 children (98.5%) were enrolled during the study period in 2014. The youngest child in the study was 9.4 months old and the oldest was 10 years old with a median age of 38 months (interquartile range: 23 to 70 months). The proportion of males in the studied population (54.1%) was higher than females (45.9%). The majority of parents had not finished high school (58.8% for fathers, 55.7% for mothers). Among the 207 children, 179 (86.5%) had lived in HCMC for at least two years. Over half of the parents reported living less than 1 km away from a CHC (56.6%). The proportion of the measles cases that occurred in persons known to have no previous measles vaccines and in those who had only one dose was 3%. The figure for unknown vaccination status was 5.%.

### Measles vaccination coverage

**Figure 1:**
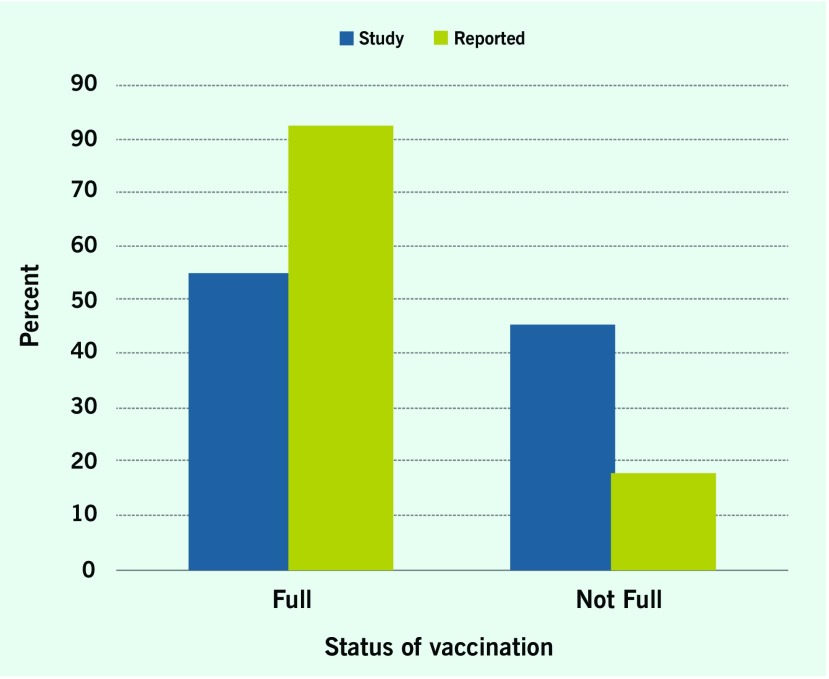
**Calculated vaccination coverage based on 2014 survey of six districts in Ho Chi Minh City, Viet Nam compared with vaccination coverage previously reported by district preventive medicine centres during 2013**

Information on vaccination status was available for 190 of the 207 children (91.8%). The parents of the remaining 17 children were uncertain of their child’s vaccination history, and their child’s vaccination cards were not available. Our survey found that the proportion of children with full vaccination coverage was 54.9% and those not fully vaccinated was 45.06%, weighted by the number of children in each age group. The proportion of children ≥ 18 months that did not have any measles vaccination was 14.8% (25/169). Consequently, there was a large vaccination gap (45.1%), which was three times higher than the vaccination gap (17.7%) reported by DPMCs (**Fig. 1**, **Table 1**).

[**insert** Figure 1]

### Epidemiological characteristics

A greater proportion of those who lived less than 1 km away were fully vaccinated, but this was not statistically significant (*p*-value = 0.7) (**Table 2**).

**Table 2:** Association between study factors and vaccination status among children aged 9 months to 10 years in six districts in Ho Chi Minh City, Viet Nam, 2014

Characteristics	*n*(%)	Fully vaccinated *n*(%)	Not fully vaccinated *n*(%)	χ^2^	*P*-value*
Age groups (*n* = 190)					
**9–18 months**	**21 (11.0)**	**13 (61.9)**	**8 (38.1)**	**0.7**	**0.4**
** ≥ 18 month–10 years**	**169 (89.0)**	**88 (52.1)**	**81 (47.9)**		
**Gender (*n* = 190)**					
**Male**	**103 (54.2)**	**55 (53.4)**	**48 (46.6)**	**0.0**	**0.9**
**Female**	**87 (45.8)**	**46 (52.9)**	**41 (47.1)**		
**Study sites (*n* = 190)**					
**District 6**	**25 (13.2)**	**15 (60.0)**	**10 (40.0)**	**9.8**	**0.1**
**Binh Chanh District**	**27 (14.2)**	**14 (51.9)**	**13 (48.2)**		
**Nha Be District**	**28 (14.7)**	**18 (64.3)**	**10 (35.7)**		
**Thu Duc District**	**28 (14.7)**	**17 (60.7)**	**11 (39.3)**		
**District 9**	**29 (15.3)**	**18 (62.1)**	**11 (37.9)**		
**District 8**	**53 (27.9)**	**19 (35.9)**	**34 (64.2)**		
**Parents’ awareness of measles vaccination (*n* = 188)**					
**Yes**	**138 (73.4)**	**72 (52.2)**	**66 (47.8)**	**0.1**	**0.8**
**No**	**50 (26.6)**	**27 (54.0)**	**23 (46.0)**		
**Father’s education (*n* = 188)**					
**Under high school**	**103 (54.8)**	**63 (61.2)**	**40 (38.8)**	**5.1**	**0.0**
**High school and above**	**85 (45.2)**	**38 (44.7)**	**47 (55.3)**		
**Mother’s education (*n* = 183)**					
**Under high school**	**100 (54.6)**	**56 (56.0)**	**44 (44.0)**	**1.1**	**0.3**
**High school and above**	**83 (45.4)**	**40 (48.2)**	**43 (51.8)**		
**Number of children under 10 years old (*n* = 190)**					
**1–2**	**173 (91.3)**	**91 (52.6)**	**82 (47.4)**	**0.2**	**0.6**
** > 2**	**17 (8.7)**	**10 (58.8)**	**7 (41.2)**		
**Distance from house to vaccination site (*n* = 188)**					
≤ **1 km**	**102 (54.3)**	**56 (55.5)**	**46 (52.9)**	**0.7**	**0.7**
**1–3 km**	**68 (36.2)**	**37 (36.4)**	**31 (35.6)**		
** > 3 km**	**18 (9.6)**	**8 (7.9)**	**10 (11.5)**		
**Causes of unvaccinated children investigated through questionnaire (*n* = 190)**					
**Fear of adverse reactions**					
**No**	**187 (98.4)**	**98 (52.4)**	**89 (47.6)**	**-**	**0.3**
**Yes**	**3 (1.6)**	**3 (100.0)**	**0 (0.0)**		
**Not old enough to vaccinate according to immunization schedule**					
**No**	**182 (95.8)**	**95 (52.2)**	**87 (47.8)**	**-**	**0.3**
**Yes**	**8 (4.2)**	**6 (75.0)**	**2 (25.0)**		
**Children’s illness**					
**No**	**182 (95.8)**	**96 (52.8)**	**86 (47.3)**	**-**	**0.5**
**Yes**	**8 (4.2)**	**5 (62.5)**	**3 (37.5)**		
**Busy parents/caregivers**					
**No**	**180 (94.7)**	**94 (52.2)**	**86 (47.8)**	**-**	**0.3**
**Yes**	**10 (5.3)**	**7 (70.0)**	**3 (30.0)**		
**Unaware of vaccination need**					
**No**	**175 (92.1)**	**92 (52.6)**	**83 (47.4)**	**0.3**	**0.6**
**Yes**	**15 (7.9)**	**9 (6.0)**	**6 (40.0)**		

* χ**2 test, Fisher’s Exact test,** *p***-value ≤ 0.05 (significance level)**

### Reasons for non-vaccination

The most common reported reason for not being fully vaccinated was the lack of awareness among parents of the need for a second dose of measles vaccine (7.9%); after their children’s first dose, the parents reported thinking that the measles vaccination process was completed. Other reasons cited for not completing the full vaccination course were: parents/caregivers were busy, parental reluctance to vaccinate children during illnesses such as the common cold, parental fear of adverse events after vaccination and children not being old enough to be vaccinated according to the immunization schedule (**Table 2**).

### Factors associated with vaccination

Children of fathers with less education (under high school versus high school and above) were more likely to be fully vaccinated (*P* < 0.05) (**Table 2**). The result of unadjusted and multiple logistic regression found that children attending study sites in districts 6 and 8 (OR = 0.49, 95% CI: 0.24–1.01) and those whose fathers had less education (OR = 0.53, 95% CI: 0.29–0.96) were less likely to be fully immunized against measles (**Table 3**).

**Table 3:** Association between study factors and vaccination status among children aged 9 months to 10 years in six districts in Ho Chi Minh City, Viet Nam, 2014: logistic regression

Predictors	Adjusted OR	95% CI	*P*-value
**Study sites**			
**District 9, Thu Duc District**	**Reference**	**-**	**-**
**Districts 6 & 8**	**0.49**	**0.24–1.01**	**0.05**
**Binh Chanh District, Nha Be District**	**0.78**	**0.89–3.62**	**0.54**
**Father’s level of education**			
**Under high school**	**Reference**	**-**	**-**
**High school and above**	**0.53**	**0.29–0.96**	**0.04**

Odds ratio (OR), 95% confidence interval (95% CI)

## Discussion

Viet Nam EPI requires the administration of the first dose of measles vaccine at 9 months of age and the second dose at 9 months after the first injection. The goal is to vaccinate at least 95% of eligible children aged 9–24 months across the country. (*18*) However, an accumulation of susceptible children throughout the years, through failure to complete full vaccine courses and incidents of vaccine failure, contributed to a gap in measles immunity in HCMC. The vaccination coverage reported by the national vaccination system in surveyed wards during the same time as our study was 82.4%. The full vaccination coverage of children in our study was only 54.9%, suggesting that the national surveillance system may be overestimating vaccination coverage. To address this issue, we strongly recommend that all children’s vaccinations should be registered in the National Immunization Information System or a digital immunization registry. (*19*, *20*) Furthermore, measles vaccination should be provided for all eligible children.

The survey showed that most parents took their children to receive the first dose of measles vaccine at 9 months old but only 52% returned for the second scheduled dose at 18 months, indicating an important but not statistically significant drop off from children receiving their first dose (62%) of measles vaccine to those receiving their second dose (52%). Other studies have found that a lack of awareness of the need for the second vaccination was associated with low coverage in this age group. A cross-sectional study in Mali showed that lack of awareness was the most common reason for non-vaccination against six diseases. (*21*) A birth cohort of 64 000 children aged 5 years old in Australia also reported that the most important reason for non-uptake of measles vaccination was lack of awareness. (*22*) However, our study found no difference in vaccination status associated with parental awareness.

We found the father’s level of education was significantly associated: children whose father had completed at least high school were less likely to be fully vaccinated compared to those whose fathers had less education. Although education levels were defined differently in our study (high school degree) compared with a study in France (bachelor’s degree), the findings were similar. (*23*) The reason for this finding has not been adequately studied, but it is possible that parents with a higher level of education may be more likely to perceive a risk of adverse side-effects or parents may have been afraid of a complete the vaccinations due to complications and high costs of vaccines. (*24*)

Children who lived in Districts 6 and 8 were less likely to be fully vaccinated compared to those living in District 9 and Thu Duc District. The difference in vaccination coverage was not significant in Binh Chanh and Nha Be districts compared to District 9 and Thu Duc District. Parents’ educational level and population fluctuations possibly account for the difference between sites. The average population change of Districts 6 and 8 was less than District 9 and Thu Duc District from 2012 to 2014. (*23*)

Limitations of this study included the lack of vaccination histories, especially with regard to the second dose. Vaccination status was based on parental recall in 8.2% of subjects, where the child’s vaccination card had been lost or health staff did not record the vaccine when it was administered. We do not have data on immigration, an important risk factor of measles transmission, so we could not take this into account when we compared vaccination coverage among communities. We do not know how many Vietnamese workers, for instance those employed in industrial parks in HCMC who came from the northern regions or the Mekong Delta, did not register their children with the national vaccination system. (*25*) Furthermore, the study selected only the youngest child instead of all children in each household, which might lead us not to have the most representative data.

## Conclusions

We found that full vaccination coverage was 67% of the vaccination coverage reported by DPMCs of the seven study wards. While 85% percent of children over 18 months had received a first dose of vaccine, the age group from 18 months to 10 years was less likely to be fully vaccinated because parents were unaware of the second measles vaccine dose at 18 months of age. Furthermore, 38% of children aged 9–18 months lacked even a first vaccination dose – a high rate of undervaccination for this age range. This highlights the critical importance of increasing first dose coverage in children from 9–18 month, and potentially in children ≥ 18 months of age. Ensuring at least one vaccination dose for children may be as important (or more) as ensuring the second vaccination dose in children over 18 months of age.

Lack of information on measles vaccination and other reasons such as children’s illness at immunization time and fear of adverse events contributed to the measles vaccination gap. Health staff should monitor actively for children who received incomplete vaccinations and schedule the second vaccination for children who have had only one dose of measles vaccination. Lack of the second vaccination dose was a key obstacle to eliminating the vaccination gap; therefore, a catch-up mass vaccination campaign should be implemented. Additionally, health promotion of measles vaccination directed towards parents would likely improve vaccination coverage.

## References

[1] Measles elimination field guide, 2013. Manila: WHO Regional Office for the Western Pacific; 2013. Available from: http://www.wpro.who.int/immunization/documents/measles_elimination_field_guide_2013.pdf

[2] Measles Is A. Killer: It Took 145,000 Lives Worldwide Last Year. National Public Radio; 2015. Available from: https://www.npr.org/2015/01/30/382716075/measles-is-a-killer-it-took-145-000-lives-worldwide-last-year, accessed on 22 Jul 2016.

[3] Gastañaduy PA, Redd SB, Fiebelkorn AP, Rota JS, Rota PA, Bellini WJ, et al.; Division of Viral Disease, National Center for Immunization and Respiratory Diseases, CDC. Measles - United States, January 1-May 23, 2014. MMWR Morb Mortal Wkly Rep. 2014 6 6;63(22):496–9. 24898167 PMC5779360

[4] Woudenberg T, van Binnendijk RS, Sanders EAM, Wallinga J, de Melker HE, Ruijs WL, et al. Large measles epidemic in the Netherlands, May 2013 to March 2014: changing epidemiology. Euro Surveill. 2017 1 19;22(3):30443. 10.2807/1560-7917.ES.2017.22.3.30443 28128092 PMC5322286

[5] Gibney KB, Brahmi A, O’Hara M, Morey R, Franklin L. Challenges in managing a school-based measles outbreak in Melbourne. Queensland: Australian and New Zealand Journal of Public Health; 2014. [cited 2019 Jul 19]. Available from: Available from https://onlinelibrary.wiley.com/doi/full/10.1111/1753-6405.12620 10.1111/1753-6405.12620 27960246

[6] Elimination CP-M. Viet Nam. Manila: WHO Regional Office for the Western Pacific; 2012Available from: Available from http://www.wpro.who.int/immunization/documents/VNM_dec2012.pdf

[7] Reported measles cases and incidence rates by WHO Member States 2013, 2014 as of 11 February 2015. Geneva: World Health Organization; 2015. Available from: https://www.who.int/immunization/monitoring_surveillance/burden/vpd/surveillance_type/active/measlesreportedcasesbycountry.pdf

[8] Urgent supports against the outbreak of measles in Viet Nam. Tokyo: Japan International Cooperation Agency; 2014. Available from: https://www.jica.go.jp/project/english/vietnam/017/news/general/140727.html, accessed on 20 Jul 2016.

[9] Measles control in Viet Nam. Manila: WHO Regional Office for the Western Pacific; 2014. Available from: http://www.wpro.who.int/vietnam/mediacentre/features/measles_control_vietnam_2014/en/, accessed on 22 Jul 2016.

[10] Phan TLNV, Ho VT, Phan CH, Vo NQ, Nguyen TPL, et al. Epidemiological characteristics of measles outbreak in southern part, Viet Nam, 2013–2014. Preventive Medicine Journals. 2014;3(152):19.

[11] Statistical yearbook of Vietnam. 2018. Hanoi: General Statistics Office Of Vietnam; 2018. Available from: https://www.gso.gov.vn/default_en.aspx?tabid=515&idmid=5&ItemID=19299, accessed on 4 Mar 2019.

[12] Weighted average. Maryland: Prince George’s Community College; 2010. Available from: http://www.pgccphy.net/ref/weightave.pdf, accessed 28 Jul 2017.

[13] Guidelines for conducting community surveys on injuries and violence. Geneva: World Health Organization; 2004. Available from: https://apps.who.int/iris/handle/10665/42975 10.1080/156609704/233/327505 15903167

[14] The 2009 Vietnam Population and Housing census: Completed results. Hanoi: General Statistics Office Of Vietnam; 2010. Available from: https://www.gso.gov.vn/default_en.aspx?tabid=515&idmid=5&ItemID=10799, accessed on 19 Jul 2019.

[15] Methods in sample surveys: cluster sampling. Maryland: Johns Hopkins University; 2009. Available from: http://ocw.jhsph.edu/courses/StatMethodsForSampleSurveys/PDFs/Lecture5.pdf, accessed on 19 Jul 2019.

[16] BMA. An R package for Bayesian Model. R News; 2005. Available from: https://www.r-project.org/doc/Rnews/Rnews_2005-2.pdf, accessed on 19 Jul 2019.

[17] Raftery AE. Bayesian model selection in social research. Sociol Methodol. 1995;25:111 10.2307/271063

[18] Measles-rubella vaccination rate reached over 95%. Hanoi: Department of Preventive Medicine, Ministry of Health; 2015. Available from: http://vncdc.gov.vn/vi/tiem-vac-xin-soi-rubella-tre-1-14-tuoi/657/ty%CC%89-le%CC%A3-tiem-chu%CC%89ng-so%CC%89i-rubella-da%CC%A3t-tren, accessed on 20 Jul 2016. Vietnamese.

[19] Health ministry launches National Immunisation Information System. Hanoi: Viet Nam News; 2017. Available from: https://vietnamnews.vn/society/health/373535/health-ministry-launches-national-immunisation-information-system.html#Krh5RJ7oXHceJws0.97, accessed on 4 Mar 2019.

[20] Nguyen NT, Vu HM, Dao SD, Tran HT, Nguyen TXC. Digital immunization registry: evidence for the impact of mHealth on enhancing the immunization system and improving immunization coverage for children under one year old in Vietnam. mHealth. 2017 7 19;3:26. 10.21037/mhealth.2017.06.03 28828373 PMC5547172

[21] Koumaré AKTD, Traore D, Haidara F, Sissoko F, Traoré I, Dramé S, et al. Evaluation of immunization coverage within the Expanded Program on Immunization in Kita Circle, Mali: a cross-sectional survey. BMC Int Health Hum Rights. 2009 10 14;9(S1) Suppl 1:S13. 10.1186/1472-698X-9-S1-S13 19828057 PMC3226232

[22] Lawrence GLMC, MacIntyre CR, Hull BP, McIntyre PB. Measles vaccination coverage among five-year-old children: implications for disease elimination in Australia. Aust N Z J Public Health. 2003;27(4):413–8. 10.1111/j.1467-842X.2003.tb00419.x 14705304

[23] Toure A, Saadatian-Elahi M, Floret D, Lina B, Casalegno J-S, Vanhems P. Knowledge and risk perception of measles and factors associated with vaccination decisions in subjects consulting university affiliated public hospitals in Lyon, France, after measles infection. Hum Vaccin Immunother. 2014;10(6):1755–61. 10.4161/hv.28486 24637343 PMC5396226

[24] Gowda C, Schaffer SE, Kopec K, Markel A, Dempsey AF. Does the relative importance of MMR vaccine concerns differ by degree of parental vaccine hesitancy?: An exploratory study. Hum Vaccin Immunother. 2013 2;9(2):430–6. 10.4161/hv.22065 23032161 PMC3859768

[25] Seo DK, Kwon Y. In-migration and housing choice in Ho Chi Minh City: Toward sustainable housing development in Vietnam. Sustainability. 2017;9(10):1738 10.3390/su9101738

[26] Sanou A, Simboro S, Kouyaté B, Dugas M, Graham J, Bibeau G. Assessment of factors associated with complete immunization coverage in children aged 12-23 months: a cross-sectional study in Nouna district, Burkina Faso. BMC Int Health Hum Rights. 2009 10 14;9(S1) Suppl 1:S10. 10.1186/1472-698X-9-S1-S10 19828054 PMC2762310

